# Theoretical Studies on the Quantitative Structure–Toxicity Relationship of Polychlorinated Biphenyl Congeners Reveal High Affinity Binding to Multiple Human Nuclear Receptors

**DOI:** 10.3390/toxics12010049

**Published:** 2024-01-08

**Authors:** Andrei Raphael M. Carrera, Elisa G. Eleazar, Alvin R. Caparanga, Lemmuel L. Tayo

**Affiliations:** 1 School of Graduate Studies, Mapúa University, Manila 1002, Philippines; armcarrera@mymail.mapua.edu.ph (A.R.M.C.); egeleazar@mapua.edu.ph (E.G.E.); 2 School of Chemical, Biological, and Materials Engineering and Sciences, Mapúa University, Manila 1002, Philippines; arcaparanga@mapua.edu.ph; 3 Department of Biology, School of Medicine and Health Sciences, Mapúa University, Makati 1200, Philippines

**Keywords:** polychlorinated biphenyls, nuclear receptors, molecular docking, molecular dynamics

## Abstract

Polychlorinated biphenyls (PCBs) are organic chemicals consisting of a biphenyl structure substituted with one to ten chlorine atoms, with 209 congeners depending on the number and position of the chlorine atoms. PCBs are widely known to be endocrine-disrupting chemicals (EDCs) and have been found to be involved in several diseases/disorders. This study takes various molecular descriptors of these PCBs (e.g., molecular weight) and toxicity endpoints as molecular activities, investigating the possibility of correlations via the quantitative structure–toxicity relationship (QSTR). This study then focuses on molecular docking and dynamics to investigate the docking behavior of the strongest-binding PCBs to nuclear receptors and compares these to the docking behavior of their natural ligands. Nuclear receptors are a family of transcription factors activated by steroid hormones, and they have been investigated to consider the impact of PCBs on humans in this context. It has been observed that the docking affinity of PCBs is comparable to that of the natural ligands, but they are inferior in terms of stability and interacting forces, as shown by the RMSD and total energy values. However, it is noted that most nuclear receptors respond to PCBs similarly to how they respond to their natural ligands—as shown in the RMSF plots—the most similar of which are seen in the ER, THR-β, and RAR-α. However, this study is performed purely in silico and will need experimental verification for validation.

## 1. Introduction

**Figure 1:**
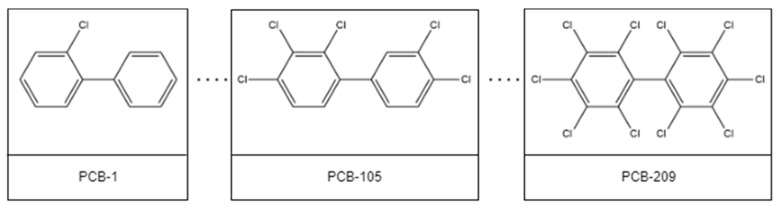
Sample PCB structures.

Polychlorinated biphenyls (PCBs) are a class of anthropogenic organic chemicals with a biphenyl structure substituted with one to ten chlorine atoms. Due to the multiple positions of substitution, there are 209 different PCBs, commonly referred to as congeners and numbered based on the amount and position of the chlorines [1]. Some are shown in Figure 1.

PCBs are known to have excellent dielectric properties, are resistant to chemical and thermal degradation, are not affected by light, and are not flammable. These properties have led to a variety of industrial and commercial applications, the most important of which is as insulating fluid in transformers and capacitors. They are also used as additives in the manufacture of paint, plastics, and carbonless copy paper [2]. However, early studies on PCBs have reported the harmful effects of these compounds on workers in factories that manufactured them [3]. Studies show that many of these hydrophobic and lipophilic compounds are highly resistant to metabolism in vertebrate species, including humans. As a result of these properties, biomagnification occurs throughout the food chain, and high tissue concentrations can often occur in top predator species [4]. In 2001, the Stockholm Convention listed PCBs as one of the persistent organic pollutants (POPs): compounds that remain intact for exceptionally long periods of time; become widely distributed throughout the environment as a result of natural processes involving soil, water, and air; accumulate in living organisms, including humans; are found at higher concentrations in the food chain; and are toxic to both humans and wildlife [5]. Despite the ban on the production and use of PCBs, inadvertent and unintentional PCBs are generated due to incomplete combustion during waste incineration or during industrial production processes. Pigment-derived PCBs can be released into the environment through different steps including pigment production, application, and disposal. They can contaminate atmospheric, terrestrial, and aquatic ecosystems, and consequently affect the inhabitant organisms [6]. This results in human exposure through food or environment and leads to a number of PCB congeners in human tissue, blood, and milk [7]. This, coupled with the persistent nature of the PCBs, requires a more elaborate study on these pollutants.

Several studies have been made on the correlation between the molecular features of substances and their biological activities. Such studies employ quantitative structure–activity relationship (QSAR), which is a modeling technique that involves constructing predictive models of biological activities as a function of structural and molecular information of a compound [8]. The concept of QSAR was initially used for drug discovery and development but has since been used to include not only biological activities, but also other physicochemical properties, including toxicity. This method is termed QSTR, quantitative structure–toxicity relationship. This technique recognizes the fact that pollutants with similar chemical structures are likely to have similar physicochemical properties and thereby exhibit equivalent toxicological behavior. QSTR models correlating the structures of 963 organic compounds to toxicity to fathead minnow were reported [9]. Toxicity to daphnia magna by agrochemicals and pesticides was studied and reported [10,11]. In terms of toxicity to rats and rodents, QSTR models were created and correlated [12,13,14,15]. Toxicity prediction models of personal care products and polychlorinated naphthalenes to algae and fish were reported by [16,17,18]. The toxicity of protection products to honey bees was studied [19]. The QSTR models reported the toxicity in terms of either pEC50 or pLD50.

Nuclear receptors play a pivotal role in regulating various physiological processes by mediating the transcriptional activity of specific genes. These receptors are intracellular proteins that bind to small molecules or ligands, and upon activation, they translocate to the cell nucleus where they modulate gene expression through interactions with DNA.

Our understanding of PCB toxicology is primarily based on in vivo and in vitro tests, which involve studying samples collected from the field [20,21]. These data usually come in the form of toxicity endpoints, examples of which are those exhibited in the US EPA TEST software (version 5.1.2) (e.g., oral rat LC_50_, *D. magna* LC_50_, fathead minnow LC_50_). Such studies, however, do not provide insight into the underlying molecular mechanisms which cause these toxicity endpoints, thus highlighting the importance of QSTR. By highlighting how certain molecules’ structures impact their biological activity, QSTR allows for further research such as drug design [22], discovery of emerging contaminants/toxicants [23], and correlation of interspecies toxicity relationships [24].

**Figure 2:**
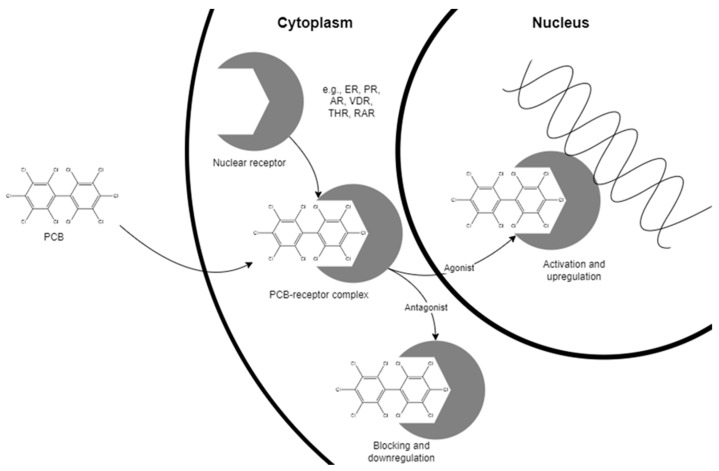
Molecular mechanism of PCB binding on nuclear receptors.

PCBs continue to be studied and revealed as carcinogenic agents for different types of cancers, such as non-Hodgkin lymphomas [25], breast cancer [26,27,28], and prostate cancer [29]. It is noted how research into the carcinogenicity of PCBs primarily focuses on their activity on the estrogen receptor due to their ability to act as structural analogues of hormones [30], especially estrogen. This makes various hormone-sensitive tissues quite susceptible to the effects of PCBs, requiring research to be conducted on the potential adverse effects on these tissues (including cancer), such as the breast [26,31,32], prostate [33,34,35], and thyroid gland [36,37,38]. Overall, their function as endocrine-disrupting chemicals (EDCs) has been highlighted and seems to be the primary driving force behind research on PCBs. Figure 2 shows the general mechanism of action of PCBs as hormonally active structural analogues, and as EDCs.

The various methodologies in this study were used to provide focus on the molecular identity and mechanisms of PCBs as EDCs, and to solely consider these molecular factors in their modes of action. To this end, molecular docking simulations of PCBs with various nuclear receptors were run, contrary to other studies which tend to focus on a single receptor or group of receptors. Such a consideration arose from the potential of PCBs to act as hormone analogues, which may not necessarily confine them to the estrogen receptor. The considered receptors include the various sex hormone/steroid receptors (estrogen, progesterone, and androgen), the vitamin D receptor, the thyroid hormone receptor (α and β), and the retinoic acid receptor (α and β). Considering these receptors allowed the study of the various intermolecular bonding forces across all receptor–ligand pairs and therefore the detection of various statistics of interest.

**Figure 3:**
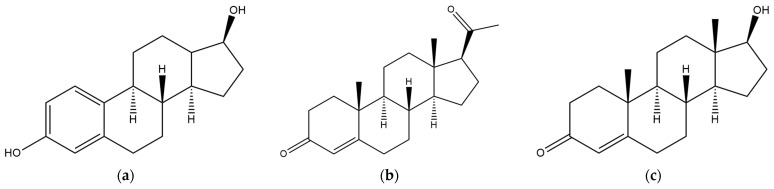
Sex hormones (**a**) estradiol, (**b**) progesterone, and (**c**) testosterone.

The establishment of the similarity of the structure of PCBs to estrogens [39] raises the question of whether PCBs will act as structural analogues of other steroid hormones as well, especially progesterone and testosterone—the most potent of the progesterone and androgen receptors, respectively, as shown in Figure 3.

**Figure 4:**
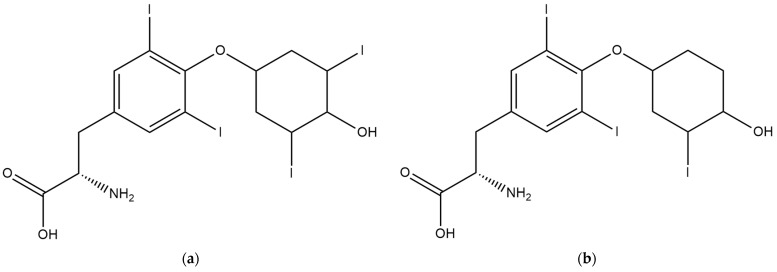
Thyroid hormones (**a**) thyroxine and (**b**) triiodothyronine.

It is also noted how PCBs may act to bind to thyroid hormone receptors due to slight similarity between the general structure of PCBs and the thyroid hormones triiodothyronine (T_3_) and thyroxine (T_4_) but not to the point of eliciting protein activity [40]. For reference, the structures of thyroxine and triiodothyronine are shown in Figure 4.

Furthermore, related pathways indicate the need to study the possibility (and mechanism, when applicable) of PCB binding to other nuclear receptors, such as the retinoic acid receptors α and β, as well as the vitamin D receptor. By investigating such interactions if and when they do exist, other effects which may not initially be thought to be caused by PCBs may be revealed—not just carcinogenicity.

While research has been conducted on the effects of certain PCB congeners and metabolites on select receptors, the molecular mechanisms of many of these interactions have yet to be fully explained, due to most of these studies showing results in need of further study.

Moreover, the wide range of physiological functions that the various nuclear receptors control indicates the need to study any molecule which may interact with them, as well as the underlying mechanisms. By understanding such mechanisms, any potential and known diseases related to such molecules can be identified, and therapies can be developed effectively.

Highlighting the physiological functions of these various nuclear receptors—as shown in Table 1—allows us to see what may result from the potential interference of EDCs; and learning the pathways and activation mechanisms allows us to hypothesize and study the manner in which EDCs may affect these receptors.

The need for a thorough systematic study of PCB effects grows as research continues to be conducted on various PCB congeners under varying conditions (e.g., model organism, geographic location). By providing a pure baseline with data derived from computational tools and studies, detection and observation of correlated parameters not included in these data could be performed, potentially opening up new avenues of research in toxicology studies.

## 2. Materials and Methods

**Scheme 1:**
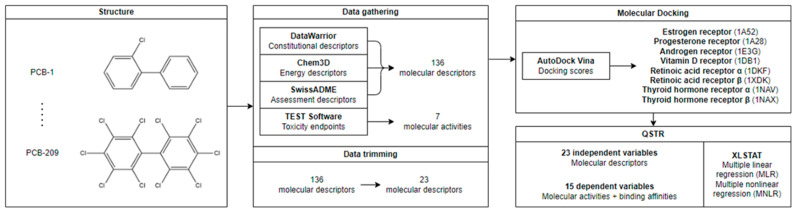
QSTR framework.

Scheme 1 presents the design of this study. The structures of PCBs (from PCB-1 to PCB-209) were constructed and used to establish a database for PCBs, allowing for the data gathering of various molecular descriptors and activities through the various software listed. This resulted in a total of 136 molecular descriptors and 7 molecular activities. However, most of the molecular descriptors were redundant and/or were identical for the whole dataset, and therefore, pre-processing and trimming of the dataset was necessary, bringing down the number of descriptors to 23. Molecular docking was then conducted to determine the predicted docking scores of each PCB congener to each human nuclear receptor investigated, coupled with molecular dynamics for investigation of these binding events. QSTR was then conducted via multiple linear regression (MLR) and multiple nonlinear regression (MNLR) to determine if there are any correlations between the molecular descriptors with the molecular activities and docking scores.

### 2.1. Data Gathering

A database of structure files for PCBs was created with the ChemDraw software (version 22.2), and the canonical SMILES format for each sketch was extracted.

Various molecular descriptors were obtained with the aid of various software for modeling of quantitative structure–activity relationships (QSARs), as shown in Table S1. Constitutional descriptors were calculated with DataWarrior [74], various molecular energies (e.g., dipole–dipole) were calculated with Chem3D, and assessment scores (e.g., synthetic accessibility, bioavailability scores) were calculated with SwissADME [75].

Alongside the molecular descriptors obtained, various molecular activities were obtained as well for QSAR modeling. The Toxicity Estimation Software Tool (TEST) from US EPA [76] was used to calculate for these molecular activities, as shown in Table S2. It is noted that the predicted toxicity endpoints match those in previous studies and databases [77,78].

### 2.2. Statistical Methods

Prior to creating a QSTR model, several variables were omitted on various bases (e.g., unchanging values), and a correlation matrix was obtained on the current variables to omit those with high correlation (R^2^ ≥ 0.90, R^2^ ≤ −0.90) to minimize redundancy. XLSTAT [79] was used for all subsequent statistical methods. The multiple linear regression (MLR) and multiple nonlinear regression (MNLR) techniques [80] were used to investigate the linear and nonlinear relationships between the calculated molecular descriptors and a selected molecular activity, and run on a training set of 189 PCBs and a test set of 20 randomly selected PCBs with the stepwise option.

Multiple nonlinear regression was conducted for all descriptor–activity pairs, with fitting for multiple models, such as polynomials (up to the power of 10), exponentials, sigmoidal and growth models, and power models, among many others, to ensure that all relationships would be caught by the regression.

Investigation of the correlation and contribution of the identified contributory descriptors in MLR was conducted via principal component analysis (PCA), which acts to decrease the dimensionality of variables, providing insight into underlying correlations.

### 2.3. Molecular Docking Simulations

Molecular docking was conducted to examine intermolecular interactions between the PCBs and target receptors, i.e., estrogen, progesterone, androgen, vitamin D, THR-α, THR-β, RAR-α, and RAR-β receptors with PDB accession codes 1A52 [81], 1A28 [82], 1E3G [83], 1DB1 [84], 1NAV [85], 1NAX [85], 1DKF [86], and 1XDK [87], respectively. BIOVIA Discovery Studio 2021 [88] was used to pre-process the receptor files (deleting water, heteroatoms, and ligands and adding polar hydrogens), and AutoGrid was then used to determine the grid box dimensions. These were set to sizes (40, 40, 40) with a spacing of 0.375 Å. Molecular docking was then conducted with AutoDock Vina [89,90], and visualizations of specific PCB–receptor interactions were then rendered via AutoDockTools.

### 2.4. Molecular Dynamics Simulations

Molecular dynamics simulations were conducted to confirm and observe the stability of protein–ligand complexes with reference ligands and PCB molecules. Only those PCBs with the best docking scores were selected for MD simulations, contrary to molecular docking, wherein all PCBs were used for the docking simulations. These simulations were conducted using GROMACS [91] using 50 ns. The output files of molecular docking were used as input files for molecular dynamics simulations, using Discovery Studio 2021 to aid in preparing the necessary files. The simulations were conducted using the CHARMM36 all-atom force field [92] and the CHARMM-modified TIP3P (transferable intermolecular interaction potential 3 points) type [93]. The models were neutralized via the addition of water molecules and counter ions as solvent, reflecting physiological conditions. The simulations allowed for the verification of possible formed bonds during binding (e.g., hydrogen bonds) via direct analysis of distance and angle between atoms, the RMSD (root mean square deviation) of the ligand to determine deviation during simulations, and the interaction energies between the receptor–ligand pair.

## 3. Results and Discussion

### 3.1. Data Gathering

Data gathering for independent variables was conducted with DataWarrior, Chem3D, and SwissADME and yielded a total of 126 molecular descriptors, trimmed down to 23 once elimination of redundant and uniform variables was made. Of the 23 descriptors, 14 were constitutional descriptors: molecular weight, Cl atoms, symmetric atoms, total surface area, globularity (SVD), globularity (volume), van der Waals surface area, van der Waals volume, shape index, molecular flexibility, molecular complexity, log P, log S, and molar refractivity. Eight were energy descriptors: stretch energies, bend energies, stretch–bend energies, torsion energies, non-1,4 van der Waals energies, 1,4 van der Waals energies, dipole–dipole energies, and total energy. Lastly, one was an assessment descriptor: synthetic accessibility.

Upon determining the molecular descriptors, calculations for the molecular activities were then made with the US EPA TEST software. These activities will serve as dependent variables in QSTR, with the goal of determining relationships and possible models which can be constructed based on the molecular descriptors. These molecular activities are developmental toxicity, bioconcentration, AMES mutagenicity, oral rat LC_50_ (48 h), *T. pyriformis* IGC_50_ (48 h), fathead minnow LC_50_ (48 h), and *D. magna* LC_50_ (48 h). The lethal and inhibition concentration values (oral rat, *T. pyriformis*, fathead minnow, *D. magna*) were also expressed in log_10_ values.

### 3.2. QSTR Models

Quantitative structure–toxicity relationship (QSTR) models are the result of the application of a similar computational modeling approach in QSAR but focused on adverse molecular activities, thereby allowing the discovery of relationships between certain molecular properties and adverse effects (e.g., molecular weight related to mutagenicity). QSTR allows the investigation of the relationship between various molecular descriptors (e.g., molecular weight) and adverse molecular activities (e.g., developmental toxicity), thereby forming quantitative models to explain how these descriptors influence molecular activities.

The MLR technique yielded three linear models:

- Bioconcentration log_10_ value (Equation (1));
- *D. magna* LC_50_ (48 h) log_10_ value (Equation (2));
- Fathead minnow LC_50_ (48 h) log_10_ value (Equation (3)).

The MNLR technique yielded two nonlinear models:

- *T. pyriformis* IGC_50_ (48 h) log_10_ value (Equation (4));
- Fathead minnow LC_50_ (48 h) value (Equation (5)).

BC log_10_ = 1.240 (7.742 × 10^−3^)MW + (8.818 × 10^−3^)SAt + (4.821 × 10^−1^)SI + (3.026 × 10^−3^)B + (3.817 × 10^−3^)T + (2.772 × 10^−1^)SAc (1) (1)

DM LC_50_ (48 h) log_10_ = (2.262 × 10^−1^) − (1.564 × 10^−2^)SAt + (2.931 × 10^−2^)TSA − (4.838 × 10^−1^)GSVD + (1.593)SI + (2.007)MF − (2.777 × 10^−2^)DD (2)

FM LC_50_ (48 h) log_10_ = 2.092 + (1.305 × 10^−2^)MW + (2.866 × 10^−3^)NVDW − (1.582 × 10^−2^)DD + (3.574 × 10^−1^)SAc (3)

TP IGC_50_ (48 h) log_10_ = -4.146 + (24.86)Cl − (28.56)Cl^2^ + (17.27)Cl^3^ − (5.964)Cl^4^ + (1.221)Cl^5^ − (1.461 × 10^−1^)Cl^6^ + (9.424 × 10^−3^)Cl^7^ − (2.524 × 10^−4^)Cl^8^ (4)

FM LC_50_ (48 h) = -799.6 + (22.56)MW − (2.697 × 10^−1^)MW^2^ + (1.798 × 10^−3^)MW^3^ − (7.347 × 10^−6^)MW^4^ + (1.888 × 10^−8^)MW^5^ − (2.987 × 10^−11^)MW^6^ + (2.662 × 10^−14^)MW^7^ − (1.024 × 10^−17^)MW^8^ (5)

Of the 23 descriptors, 12 descriptors in total were found to be correlated and thus were able to generate models. The descriptors for each MLR model are shown in Table 2.

Of the total 11 correlated descriptors for MLR models, 6 were constitutional descriptors: molecular weight (MW), which is the sum of the atomic weights of atoms in a molecule; symmetric atoms (SAt), which counts the number of symmetric atoms across rings (e.g., ortho, meta); total surface area (TSA), which is the sum of all atomic surfaces of all atoms of a molecule; globularity (SVD), which is the measure of molecular globularity via single value decomposition; shape index (SI), which is a measure involving the distance and number of atoms in a molecule; and molecular flexibility (MF), which involves the number of rotatable bonds and their contribution to the overall flexibility of a molecule. Four of the total correlated descriptors were energy descriptors: bend energies (B), which represent energies associated with bond angles away from their optimal values; torsion energies (T), which represent energies associated with bonds deformed torsionally; non-1,4 VDW energies (NVDW), which represent energies associated with interactions between atoms separated by more than three atoms; and dipole–dipole energies (DD), which represent energies associated with interactions of bond dipoles. The last descriptor was an assessment descriptor: synthetic accessibility (SAc), determined by SwissADME as a measure of the ease of chemical synthesis, ranging from 1 (easiest) to 10 (hardest).

**Figure 5:**
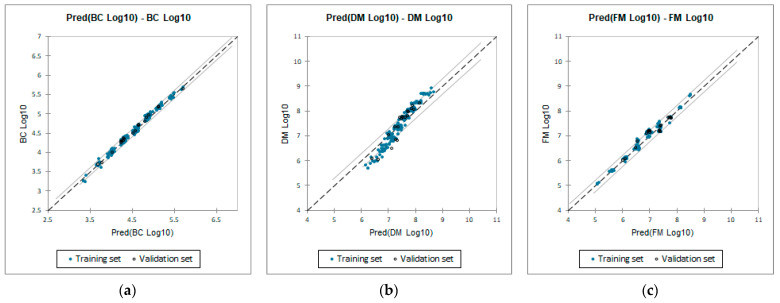
Validation for MLR models: (**a**) bioconcentration log_10_, (**b**) *D. magna* LC_50_ (48 h) log_10_, and (**c**) fathead minnow LC_50_ (48 h) log_10_.

**Figure 6:**
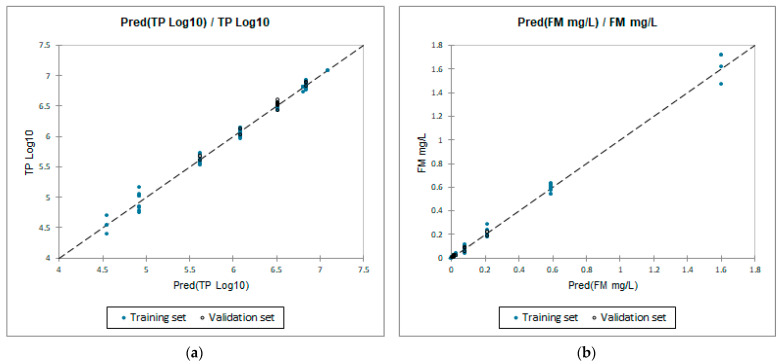
Validation for MNLR models: (**a**) *T. pyriformis* IGC_50_ (48 h) log_10_ and (**b**) fathead minnow LC_50_ (48 h).

All constructed models reached a favorable goodness-of-fit score of R^2^ ≥ 0.95, and the ANOVA tests between the training and validation sets all had favorable results (<0.0001). The validation models are shown in Figure 5 and Figure 6, showing the comparison of the actual and predicted values of the training and validation sets for each model.

**Figure 7:**
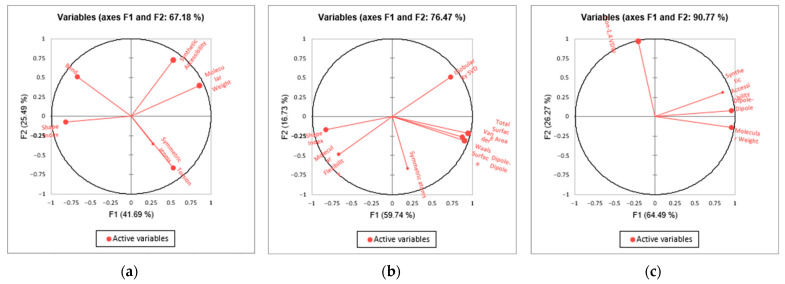
PCA biplots of MLR models: (**a**) bioconcentration log_10_, (**b**) *D. magna* LC_50_ (48 h) log_10_, and (**c**) fathead minnow LC_50_ (48 h) log_10_.

PCA was then conducted to allow for analysis of the correlation between the correlated descriptors within models, made possible by reducing the dimensionality of the variables and portraying them in PCA biplots. The PCAs of the MLR models are shown in Figure 7, showing correlations between contributory descriptors for each model.

Figure 7a shows a high correlation between the number of symmetric atoms and torsion energies, at which the relationship arises when an increase in symmetric atoms at the 2/2′ and 6/6′ positions result in the PCB twisting to dissipate energies. It can also be seen that there is some correlation between the molecular weight and synthetic accessibility, signifying that a higher molecular weight of PCBs results in a more difficult synthesis pathway. Lastly, there is a slight correlation between the shape index and bend energies.

Figure 7b shows a high correlation between the total surface area, the van der Waals surface area, and dipole–dipole energies, meaning that as the total surface area increases (solely due to addition of chlorine atoms), the van der Waals surface area and dipole–dipole energies increase as well. A slight correlation can also be seen between the shape index and molecular flexibility.

Lastly, Figure 7c shows a high correlation between the molecular weight, synthetic accessibility, and dipole–dipole energies, which all increase alongside each other to some extent, but not so highly correlated with each other as to be omitted.

### 3.3. Molecular Docking

**Figure 8:**
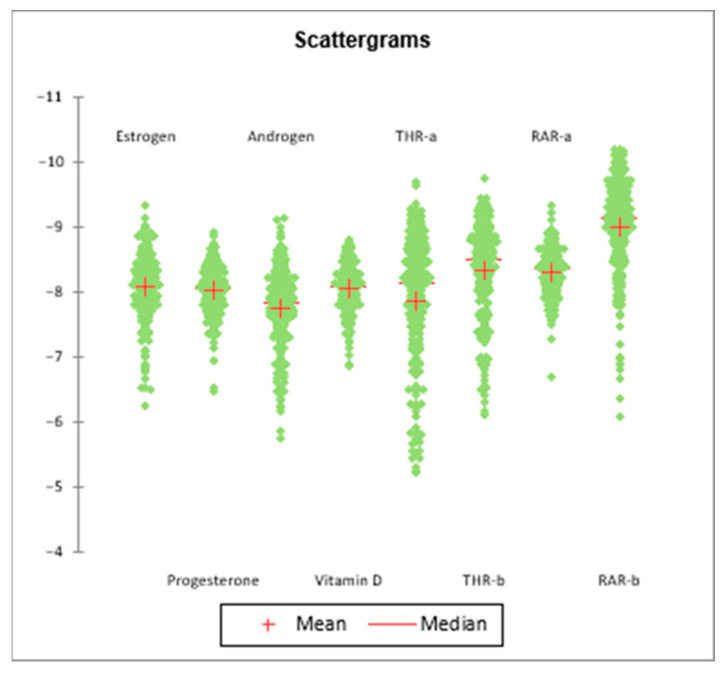
Scattergrams of molecular docking results.

Molecular docking was then conducted to analyze the relative docking scores of the PCB group with various nuclear receptors. While it may be seen whether PCBs bind more strongly to some receptors than others, this does not give insight into the resulting effect—whether the PCB functions as an antagonist or not. Figure 8 shows the distributions of the docking scores of PCBs of eight nuclear receptors.

**Figure 9:**
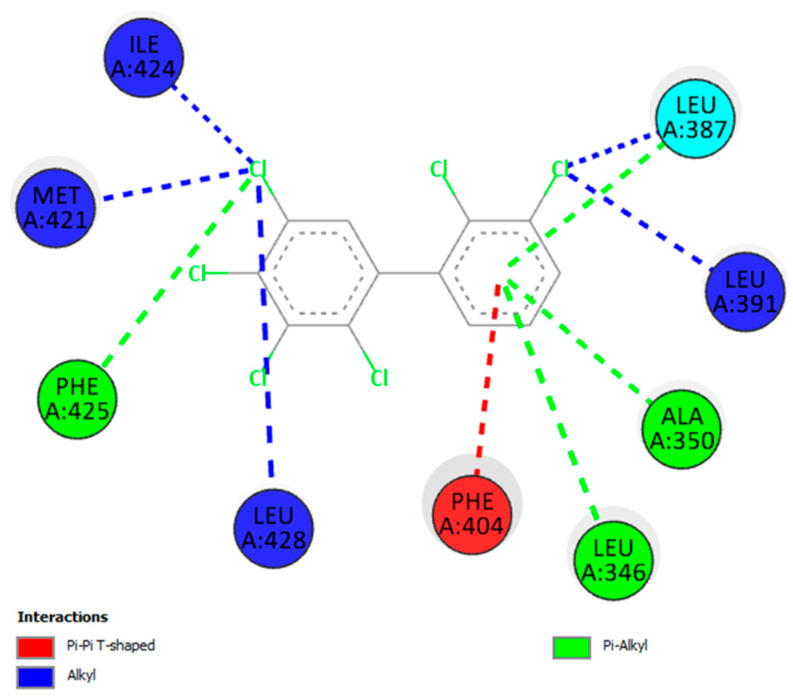
Amino acid interactions of PCB-129 with the estrogen receptor.

The maximum score observed is in retinoic acid receptor β (~−10 kcal/mol), whereas the weakest docking score observed is in thyroid hormone receptor α (~−5.2 kcal/mol). It is also worth noting that the distribution of the docking scores in thyroid hormone receptor α is over a large range (−5.2–−9.7 kcal/mol), whereas the distribution of docking scores in the vitamin D receptor is the most focused (−6.9–−8.8 kcal/mol). Visualizations were extracted from the top PCBs with the strongest docking scores for further analysis, as shown in Figure 9 and in Table 3. While other binding modes were observed, the determined optimal binding mode was taken on the basis of consistency of pose and interacting residues.

All of the interactions presented in Figure 9 are hydrophobic; this is true for most PCB binding with human nuclear receptors. These hydrophobic interactions may come in the form of pi–pi T-shaped interactions (also known as CH–pi hydrogen interactions), alkyl interactions (between alkyl groups of both ligand and protein), and pi–alkyl interactions (between an alkyl group and a pi–orbital group).

Two PCBs are notable in Table 3: PCB-129 and PCB-156. Of the 209 PCBs, PCB-129 binds strongest to the estrogen receptor and second strongest to the progesterone receptor; and PCB-156 binds strongest to retinoic acid receptor β and second strongest to thyroid hormone receptor α and the androgen receptor.

**Figure 10:**
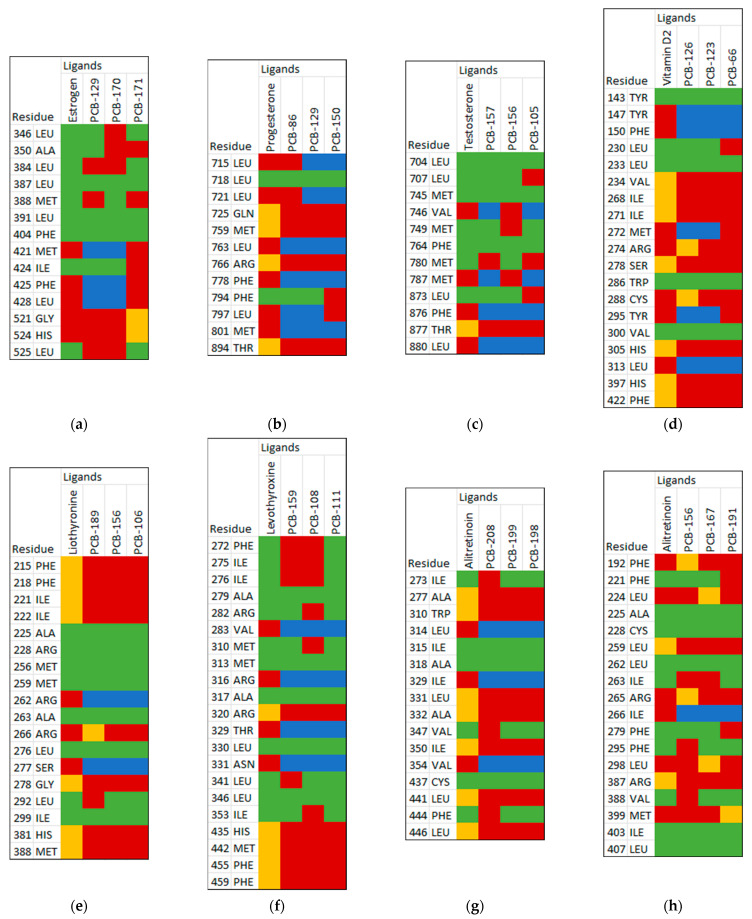
Comparison of involved amino acid sequences for (**a**) ER, (**b**) PR, (**c**) AR, (**d**) VDR, (**e**) THR-α, (**f**) THR-β, (**g**) RAR-α, and (**h**) RAR-β.

Figure 10 shows a comparison of involved amino acid sequences between the natural ligand and the top 3 PCBs with the highest docking scores.

### 3.4. Molecular Dynamics

The molecular dynamics simulations were able to shed light on the interaction dynamics of the various receptor–ligand pairs, analyzed during 50 ns for the native ligand and the strongest-binding PCB. One such feature is the root mean square deviation (RMSD), of which the final values (at the end of 50 ns) are shown in Table 4.

For the RMSD, the ligand was considered alongside the heavy atoms with reference to the protein backbone for a more accurate observation of the ligand stability. In the table, it can be seen that the natural ligands have very favorable RMSD values (<1 Å), showing that all of these natural ligands do not dissociate from their receptors at all. However, for the strongest-binding PCBs,

- Three of them have extremely favorable (<1 Å) RMSD values;
- Three of them have acceptable (1–3 Å) RMSD values; and
- Two of them have unfavorable (>3 Å) RMSD values.

Therefore, on the basis of RMSD alone, it can be inferred that while some PCBs may have higher docking scores compared to the natural ligands, the stability of such docking events is inferior.

**Figure 11:**
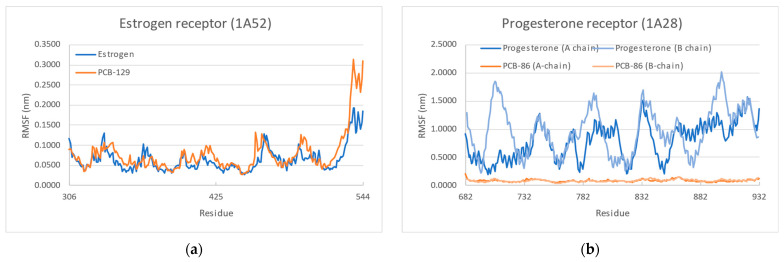

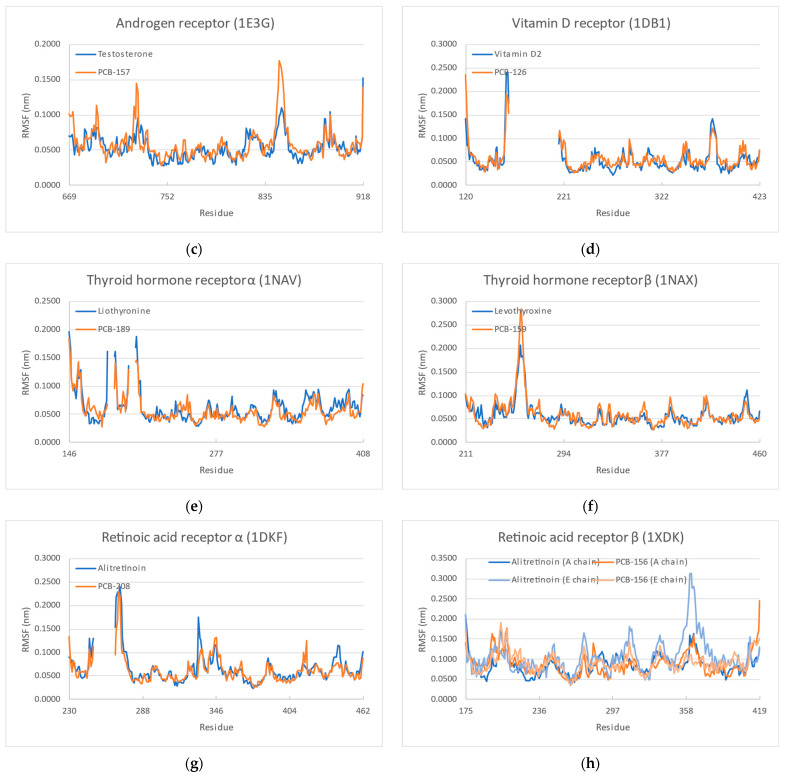
RMSF plots of the (**a**) ER, (**b**) PR, (**c**) AR, (**d**) VDR, (**e**) THR-α, (**f**) THR-β, (**g**) RAR-α, and (**h**) RAR-β.

A feature to observe alongside RMSD is the root mean square fluctuation (RMSF), which shows the fluctuation of each residue in the receptor during binding. The RMSF values were observed over the span of 50 ns and are shown in Figure 11.

For some of the receptors, there are gaps in residue numbering in the references—albeit being complete proteins—and they have been preserved in this study. In the plots, it can be seen that most nuclear receptors had similar fluctuations between their native ligands and strongest-binding PCBs, except for the PR, wherein binding of the PCB induced almost negligible fluctuations. Such similar fluctuations indicate that upon PCB binding, some conformational change occurs in the studied nuclear receptors.

Another property observed in molecular dynamics simulations is the interacting energies, the total values of which were determined over the span of 50 ns. These are shown in Table 5.

For all receptors, the native ligand exhibits a higher total interacting energy when compared to the strongest-binding PCB. This means that despite the PCB binding to certain nuclear receptors, the native ligand will still exhibit stronger binding and therefore a more secure bond.

A comparison of the docking scores of the strongest-binding PCBs and the natural ligands of the nuclear receptors under study is shown in Table 6, where we expect the natural ligands to be naturally stronger in binding than PCBs.

In Table 6, it can be seen that only three nuclear receptors have natural ligands that bind stronger than the strongest-binding PCBs (estrogen, progesterone, and vitamin D receptors). The rest of the receptors, however, have their natural ligands overpowered by the strongest-binding PCBs (androgen, THRα and β, RARα and β). From these results, it can be hypothesized that for most nuclear receptors, PCBs will—once bound—bind stronger than their natural ligand, effectively blocking access (not considering various transport concerns). However, for all PCBs, it was observed that only one binding mode was found to bind significantly,

In the molecular dynamics results, it is observed that all natural ligands have relatively better binding dynamics when compared to the strongest-binding PCB. Initially considering the root mean square deviation (RMSD) between these two molecule groups allows us to see that some of the PCBs with the strongest binding energies determined in molecular docking—such as PCB-156 with −10.190 kcal/mol when docked with RAR-β—are considerably unstable. This is observed for PCB binding to PR and RAR-β. However, all the other receptors have favorable RMSD values with their strongest-binding PCBs, albeit not as stable or secure as the natural ligand itself.

Moreover, it is also observed that the RMSF values of the two molecule groups are almost similar to each other, suggesting activation of the receptor when interacting with the strongest-binding PCB. This is observed for all but the PR, which, as noted earlier, has a considerably unfavorable RMSD value. While the RMSF plot of the PR is quite easy to interpret, the binding of the strongest-binding PCB of RAR-β is still quite similar to that of the natural ligand, but to a lesser degree when compared to the other receptors. Additionally, when observing receptors wherein the strongest-binding PCB and the natural ligands had similar RMSF plots, it is seen that while the PCB followed the general trend of fluctuation with that of the natural ligand, it was not perfectly emulated, which is to be expected due to the size difference between the molecules in these two groups. However, some receptors exhibited very similar RMSF plots between these two groups, which are the ER, THR-β, and RAR-α. This suggests that the binding of the strongest-binding PCBs to these receptors would trigger a very similar response to that of natural ligand binding, and is highly likely to correspond to activation.

Lastly, the total interacting energies are observed. The interacting energies do not denote binding energies of the ligands to the receptors but are related. The interacting energies are the collective sum of the forces between the ligand and the receptor, and highly depend on how much of the receptor is ‘in contact’ with the ligand. While the PCBs may exhibit stronger binding energies due to the confinement of smaller molecules, this does not ensure that the receptor interacts with the PCB as strongly or as securely as its natural ligand. Here, it is observed that all natural ligands have stronger interacting energies when compared to the strongest-binding PCBs, signifying a more secure binding—especially when taken alongside the RMSD values.

## 4. Conclusions

In essence, the various simulations conducted show that PCBs—even in their unmetabolized state—will bind to various nuclear receptors. Combined with various studies showing correlations of PCBs chronically affecting bodily processes (e.g., pregnancy), this highlights the importance of studying the long-term effects of EDCs such as PCBs on various other processes which may be dysregulated due to such binding activities.

It has been shown that the various toxicity endpoints of PCBs can be modeled with regards to their structural information (as shown in Equations (1)–(5)), and that such PCBs will bind to human nuclear receptors in silico. However, despite the fact that PCBs themselves will have docking scores comparable to the natural ligands (Table 6), simulations have shown that the interaction and stability of PCBs with nuclear receptors are inferior to the natural ligands (as shown in Table 4 and Table 5 and Figure 10). This, however, does not mean that they will not have an effect. Other studies have repeatedly shown a correlation between PCB exposure and disease/disorder development, thereby prompting further study.

It is important to note that in this study, data were obtained purely in silico and will require experimental testing for validation. Additionally, the metabolization of PCBs within the body leading to their transformation was not considered; this is undoubtedly an important point to consider in future studies.

## Figures and Tables

**Table 1:** Summary of physiological functions of various nuclear receptors.

Nuclear Receptor	Physiological Function	Dysfunction
Estrogen receptor	Bone health maintenance [41] Secondary sex characteristic development Cardiovascular regulation [42] Pregnancy support [43,44] Menstrual cycle support [45,46]	Breast and ovarian cancers [47]
Progesterone receptor	Breast development [47] Menstrual cycle regulation [48] Pregnancy regulation [49]	Endometriosis and infertility [50]
Androgen receptor	Facial and body hair growth [51] Muscle development [52] Voice deepening [53] Bone health maintenance [54]	Androgen insensitivity syndrome and prostate cancer [55]
Vitamin D receptor	Mineral metabolism [56] Immune response regulation [57] Inorganic phosphate homeostasis [58] Calcium homeostasis [59] Bone health maintenance [60]	Autoimmune diseases, cancer, and cardiovascular disorders [61]
Thyroid hormone receptor	Development regulation [62] Heart regulation [63] Metabolism regulation [64] Lipid metabolism [65]	Thyroid hormone resistance Hypo- and hyperthyroidism [66]
Retinoic acid receptor	Embryonic development [67] Stem cell differentiation [68] Organ development [67] Vision regulation [69] Immune regulation [70] Skin regulation [71]	Lung and breast cancer [72] Congenital malformations and skin diseases [73]

**Table 2:** Constructed MLR models and correlated descriptors.

Correlated Descriptor	Model		
	(1)	(2)	(3)
Molecular weight	✓		✓
Symmetric atoms	✓	✓	
Total surface area		✓	
Globularity (SVD)		✓	
Shape index	✓	✓	
Molecular flexibility		✓	
Bend energies	✓		
Torsion energies	✓		
Non-1,4 VDW energies			✓
Dipole–dipole energies		✓	✓
Synthetic accessibility	✓		✓
correlated descriptors	6	6	4

**Table 3:** Summary of binding interactions.

Receptor	Ligand	Binding (kcal/mol)	Interactions *					
			HP	HB	H	S	H/HB	E
Estrogen	PCB-129	−9.319	10	0	0	0	0	0
	PCB-170	−9.121	11	0	0	0	0	0
	PCB-171	−9.022	8	0	2	0	0	0
Progesterone	PCB-86	−8.921	8	0	0	0	0	0
	PCB-129	−8.898	10	0	0	0	0	0
	PCB-150	−8.839	8	0	0	0	0	0
Androgen	PCB-157	−9.121	12	0	1	0	0	0
	PCB-156	−9.102	11	0	1	0	0	0
	PCB-105	−8.999	14	0	0	0	0	0
Vitamin D	PCB-126	−8.802	18	0	0	1	0	0
	PCB-123	−8.776	13	0	0	0	0	0
	PCB-66	−8.762	12	0	0	0	0	0
THR-α	PCB-189	−9.693	16	1	1	0	0	0
	PCB-156	−9.620	15	3	0	0	0	0
	PCB-106	−9.342	15	2	0	0	0	0
THR-β	PCB-159	−9.744	17	2	1	1	0	1
	PCB-108	−9.424	17	2	1	1	0	0
	PCB-111	−9.422	21	2	1	1	0	0
RAR-α	PCB-208	−9.334	8	1	0	1	0	0
	PCB-199	−9.220	11	1	0	1	0	0
	PCB-198	−9.097	7	1	0	1	0	0
THR-β	PCB-156	−10.190	17	0	0	0	0	0
	PCB-167	−10.180	20	0	0	0	0	0
	PCB-191	−10.160	17	0	1	0	1	0

* HP—hydrophobic; HB—hydrogen bond; H—halogen; S—sulfur; H/HB—halogen/hydrogen bond; E—electrostatic.

**Table 4:** RMSD of various receptor–ligand pairs.

Receptor	Ligand	RMSD (Å)
1A52 Estrogen receptor	Estrogen	0.867
	PCB-129	1.770
1A28 Progesterone receptor	Progesterone	0.688
	PCB-86	72.702
1E3G Androgen receptor	Testosterone	0.822
	PCB-157	1.514
1DB1 Vitamin D receptor	Vitamin D2	0.245
	PCB-126	0.949
1NAV Thyroid hormone receptor α	Liothyronine (T_3_)	0.750
	PCB-189	0.863
1NAX Thyroid hormone receptor β	Levothyroxine (T_4_)	0.366
	PCB-159	0.736
1DKF Retinoic acid receptor α	Alitretinoin	0.902
	PCB-208	2.076
1XDK Retinoic acid receptor β	Alitretinoin	0.051
	PCB-156	27.997

**Table 5:** Interacting energies for each receptor–ligand pair.

Receptor	Ligand	Total Interacting Energy (kcal/mol)
1A52 Estrogen receptor	Estrogen	55.576 ± 3.754
	PCB-129	38.714 ± 1.981
1A28 Progesterone receptor	Progesterone	50.825 ± 3.743
	PCB-86	31.388 ± 2.778
1E3G Androgen receptor	Testosterone	42.381 ± 1.412
	PCB-157	37.717 ± 2.911
1DB1 Vitamin D receptor	Vitamin D2	61.509 ± 3.064
	PCB-126	36.917 ± 1.386
1NAV Thyroid hormone receptor α	Liothyronine (T_3_)	64.077 ± 3.703
	PCB-189	49.320 ± 2.681
1NAX Thyroid hormone receptor β	Levothyroxine (T_4_)	59.820 ± 4.351
	PCB-159	45.184 ± 1.475
1DKF Retinoic acid receptor α	Alitretinoin	40.208 ± 2.010
	PCB-208	38.392 ± 2.708
1XDK Retinoic acid receptor β	Alitretinoin	53.306 ± 3.258
	PCB-156	37.790 ± 2.444

**Table 6:** Docking score comparison of strongest-binding PCBs and natural ligands.

Nuclear Receptor	Natural Ligand		PCB	
	Name	Docking Score	Name	Docking Score
ER	Estradiol	−10.700	PCB-129	−9.319
PR	Progesterone	−11.450	PCB-86	−8.921
AR	Testosterone	−8.289	PCB-157	−9.121
VDR	Vitamin D2	−12.220	PCB-126	−8.802
THRα	T_3_	−9.086	PCB-189	−9.693
THRβ	T_3_	−6.649	PCB-159	−9.744
RARα	Tretinoin	−6.925	PCB-208	−9.334
RARβ	Tretinoin	−6.925	PCB-156	−10.190

## Data Availability

Data are contained within the article or in the Supplementary Materials.

## Supplementary Materials

The following supporting information can be downloaded at: https://www.mdpi.com/article/10.3390/toxics12010049/s1, Table S1.a: Molecular descriptors of PCBs; Table S1.b: Molecular activities of PCBs; Table S2.a: Docking scores of natural ligands; Table S2.b: Docking scores of PCBs.
